# Glutamatergic system components as potential biomarkers and therapeutic targets in cancer in non-neural organs

**DOI:** 10.3389/fendo.2022.1029210

**Published:** 2022-11-15

**Authors:** Ana Cristina García-Gaytán, Andy Hernández-Abrego, Mauricio Díaz-Muñoz, Isabel Méndez

**Affiliations:** Instituto de Neurobiología, Departamento de Neurobiología Celular y Molecular, Universidad Nacional Autónoma de México (UNAM), Campus UNAM-Juriquilla, Querétaro, Mexico

**Keywords:** ionotropic glutamate receptor (iGluR), metabotropic glutamate receptor (mGluR), excitatory amino acid transporter (EAAT), glutamate/cystine antiporter (xCT), cancer, non-neural organs

## Abstract

Glutamate is one of the most abundant amino acids in the blood. Besides its role as a neurotransmitter in the brain, it is a key substrate in several metabolic pathways and a primary messenger that acts through its receptors outside the central nervous system (CNS). The two main types of glutamate receptors, ionotropic and metabotropic, are well characterized in CNS and have been recently analyzed for their roles in non-neural organs. Glutamate receptor expression may be particularly important for tumor growth in organs with high concentrations of glutamate and might also influence the propensity of such tumors to set metastases in glutamate-rich organs, such as the liver. The study of glutamate transporters has also acquired relevance in the physiology and pathologies outside the CNS, especially in the field of cancer research. In this review, we address the recent findings about the expression of glutamatergic system components, such as receptors and transporters, their role in the physiology and pathology of cancer in non-neural organs, and their possible use as biomarkers and therapeutic targets.

## 1 Introduction

Glutamatergic system components are located in neural and non-neural cells. Recent studies have demonstrated that ionotropic and metabotropic glutamate receptors, along with membrane glutamate transporters, regulate a broad variety of processes in the pathophysiology of non-neural diseases, especially those related to oncologic and inflammatory conditions, highlighting their potential use as biomarkers and/or therapeutic targets. This review aims to provide an overview of recent progress in the study of glutamatergic system components and their function in cancer of non-neural organs.

## 2 Glutamate as a metabolic intermediate

Metabolic networks are complex systems that make coordinated biochemical and energy transformations possible among and within a variety of cellular compartments. In this context, some molecules, including glutamate, play important nodal roles in strategic pathways involving the concerted interactions of dozens of different intermediates.

Glutamate (2-aminopentanedioic acid) is a non-essential α-amino acid that is recognized as one of the most abundant biomolecules in cellular systems. It displays properties according to the reactivity of molecule at the tautomeric 2 carbon and at the 4- and 5-C positions, making it serve as a metabolic intermediate. The 5-carbon skeleton of its structure allows the keto-enol tautomers and transient, joint-electron activation states at the 2,3- and 4,5-bounds in positions of enzymatic catalysis. Glutamate is present in prokaryotes and along the entire phylogenetic scale, and it can be formed in prebiotic conditions from simpler entities such as HCN, NH_4_^+,^ and some aldehydes (1). As a relevant prebiotic reaction, glutamate can be oxidatively decarboxylated by sunlight photolysis towards succinate (2). Glutamate acts as a link between energy and nitrogen metabolism, and it is a key intermediate between anabolic and catabolic transformations. Glutamate functions as a 1) substrate for protein synthesis, 2) a component of the rigid β-sheet structure enriched in polyglutamate, 3) a precursor of ornithine and proline, 4) a part of the arginine cycle, 5) an inhibitor of the glutaminase reaction, 6) an energy source for some tissues (mucosa), 7) a substrate for vitamin k-dependent carboxylation of clotting factors (with high Ca^2+^ affinity), and 8) a precursor of γ-aminobutyrate (GABA; neurotransmitter and osmolyte) and glutathione (for review see (3)).

### 2.1 Glutamate and nitrogen metabolism

Nitrogen is a key element in the structure of biomolecules especially peptides and nucleic acids. To maintain nitrogen homeostasis in the brain and the whole organism, glutamate is required, functioning as a substrate and a product of several enzymes. For example, transaminases, a family of pyridoxal phosphate-dependent enzymes (aminotransferases), usually catalyze reversible reactions in which two types of conversion are possible: 1) most amino acids can transfer their amino group to α-ketoglutarate and converge in the formation of glutamate; 2) glutamate can donate its amino group to a variety of keto acids to favor the formation of the any corresponding amino acid (4). In an ATP-dependent reaction, glutamate and NH_4_^+^ are condensed to form glutamine by glutamine synthetase (GS). GS is an oligomeric enzyme expressed in the liver (exclusively in pericentral hepatocytes) and in the brain, primarily in astrocytes (5). During hyperammonemia, production of glutamine from glutamate is significantly favored. In another reaction, glutamate donates NH_4_^+^ by mitochondrial oxidative deamination in a reversible redox reaction catalyzed by glutamate dehydrogenase (GDH). In the NH_4_^+^-resulting reaction, α-ketoglutarate is formed and NAD^+^ is reduced to NADH. NADPH is oxidized in the condensation of α-ketoglutarate, derived from the Krebs cycle (6) and NH_4_^+^ to glutamate (7). Because of this biochemical equilibrium between glutamate and NH_4_^+^, in hyperammonemia conditions, such as those associated with hepatic or kidney disorders, glutamate metabolism acts as a pathogenic factor (8). Urea is the major nitrogenous excretory product in mammals; it is synthesized in the liver and is part of a key process to maintain nitrogen balance. NH_4_^+^ produced by oxidative deamination is a substrate of the urea cycle rate-limiting enzyme, carbamoyl phosphate synthetase I, and in an ATP-dependent reaction, it reacts with HCO_3_^-^ to form carbamoyl phosphate (9). In the brain, the main pathway for glutamate and ammonia removal is by GS and by glutamate decarboxylase action to produce the inhibitory neurotransmitter GABA (10).

### 2.2 Glutamate and energy metabolism

Glutamate is the most abundant amino acid in the brain with concentrations in the range of 10-100 mM within synaptosomal vesicles. Since glutamate is a major excitatory neurotransmitter, its concentration in the cerebral extracellular fluid must be kept at low micromole levels (0.5–5 μM) (11). A large proportion of cerebral glutamate is synthesized mainly *via* glutaminase reaction and by aminotransferases, and to a lesser extent by GDH reaction (12). The carbon skeleton of the glutamate molecule is formed within the mitochondria as a cataplerotic reaction based on the incorporation of an NH_4_^+^ molecule to α-ketoglutarate derived from the Krebs cycle. This pathway was also described in the liver but with less efficacy (6). GDH activity is favored towards the excision of glutamate mainly by enhancing its phosphorylation and reducing ADP-ribosylation and lysine acetylation. While NH_4_^+^ derived from this reaction is channeled to urea synthesis, α-ketoglutarate turns into gluconeogenic intermediates, mainly oxaloacetate, which is decarboxylated by PEPCK (phosphoenolpyruvate carboxykinase). In this way, both ATP-dependent metabolic pathways combine transit among organelles, as well as redox requirements, to accomplish nitrogen disposal and synthesis of energy molecules (13).

Glutamate also plays a role in the malate-aspartate shuttle, which is a link between cytosolic redox reactions with mitochondrial electron transport and oxidative phosphorylation to generate energy. Since mitochondria are impermeable to glycolytic NADH, the reducing equivalent is introduced into the mitochondria by a set of coupled redox reactions: 1) cytosolic malate dehydrogenase forms malate from oxaloacetate (in parallel, NADH is oxidized to NAD^+^); 2) malate enters the mitochondria *via* an antiporter in exchange for α-ketoglutarate; 3) mitochondrial malate dehydrogenase restitutes NADH that is available for electron transport chain (in parallel, oxaloacetate is formed); 4) glutamate allows the transamination of oxaloacetate to aspartate; 5) aspartate goes to the cytosol *via* an antiporter in exchange for cytosolic glutamate; 6) cytosolic transaminase regenerates oxaloacetate and glutamate to complete the cycle. Shortage of cytosolic glutamate may impair the function of the malate-aspartate shuttle (14).

### 2.3 Glutamate metabolism in cancer

Cancer is a collection of pathologies characterized by loss of genetic and metabolic control set-points. A set of hallmarks have been postulated as the principal feature that distinguishes cancerous cells from non-cancerous cells (15). Among the most accepted cancerogenic elements are metabolic rewiring, immune modulation, altered stress response, invasion and metastasis, vascularization, selective growth and proliferative advantage, and an inciting microenvironment (16).

Reprogrammed metabolism allows cancerous cells to sustain rapid proliferation. Two central pathways that permit biochemical adaptations of cancer metabolism are increased glycolysis and glutamine catabolism (17). These biochemical characteristics are part of a metabolic modulation that has incidence in the transit of metabolites towards and from the mitochondria. Despite oxygen availability, cancerous cells display intense glucose consumption and lactate production, known as the Warburg effect; this enhanced glycolytic activity promotes sufficient energy as ATP, coupled with glutamine metabolism, to support cellular sustainability. At the same time, substrates from the Krebs cycle, such as citrate, escape from the mitochondria to serve as precursors for the synthesis of fatty acids and generation of reducing power as NADPH (18).

Glutamine regulation is tightly controlled by metabolic intermediates that allosterically inhibit and activate GDH, which fuels the Krebs cycle by converting glutamine-derived glutamate to α-ketoglutarate. The result of this metabolic reprogramming is that mitochondria contribute with biosynthetic intermediates for membrane synthesis and DNA formation, allowing NADPH availability. At the same time, this organelle uptakes glutamine and glutamate carbon skeletons to sustain its energy generation. The coordinated response in oncogenic conditions between oxidative glycolysis and glutaminolysis involves synergistic regulation of several genes encoding metabolic enzymes, including lactate dehydrogenase isoforms A (LDHA) and B (LDHB), and mitochondrial glutamic pyruvate transaminase 2 (19).

In addition to its multiple metabolic roles, glutamate also forms part of a signaling system, acting as a specific ligand for an array of ionotropic and metabotropic receptors (20, 21).

## 3 Glutamate signaling in cancer of non-neural organs

Outside the neural environment, glutamate functions as a signaling molecule. Over the last 20 years, investigators have dedicated their research to discovering the presence of glutamatergic system components in non-neural organs. Several studies indicate an aberrant expression of glutamate receptors and/or transporters in the pathogenesis of cancer cells of non-neural organs. Diverse non-neural cancer cells have been shown to release elevated levels of glutamate, suggesting a putative autocrine loop of glutamatergic signaling (22, 23).

### 3.1 Glutamate receptors

Glutamate mediates cellular responses *via* the interaction with receptors in the plasmatic membranes of target cells. Glutamate acts *via* two classes of receptors: ion channels named ionotropic glutamate receptors (iGluRs) and G protein-coupled receptors named metabotropic glutamate receptors (mGluRs).

#### 3.1.1 Ionotropic glutamate receptors

**Figure 1:**
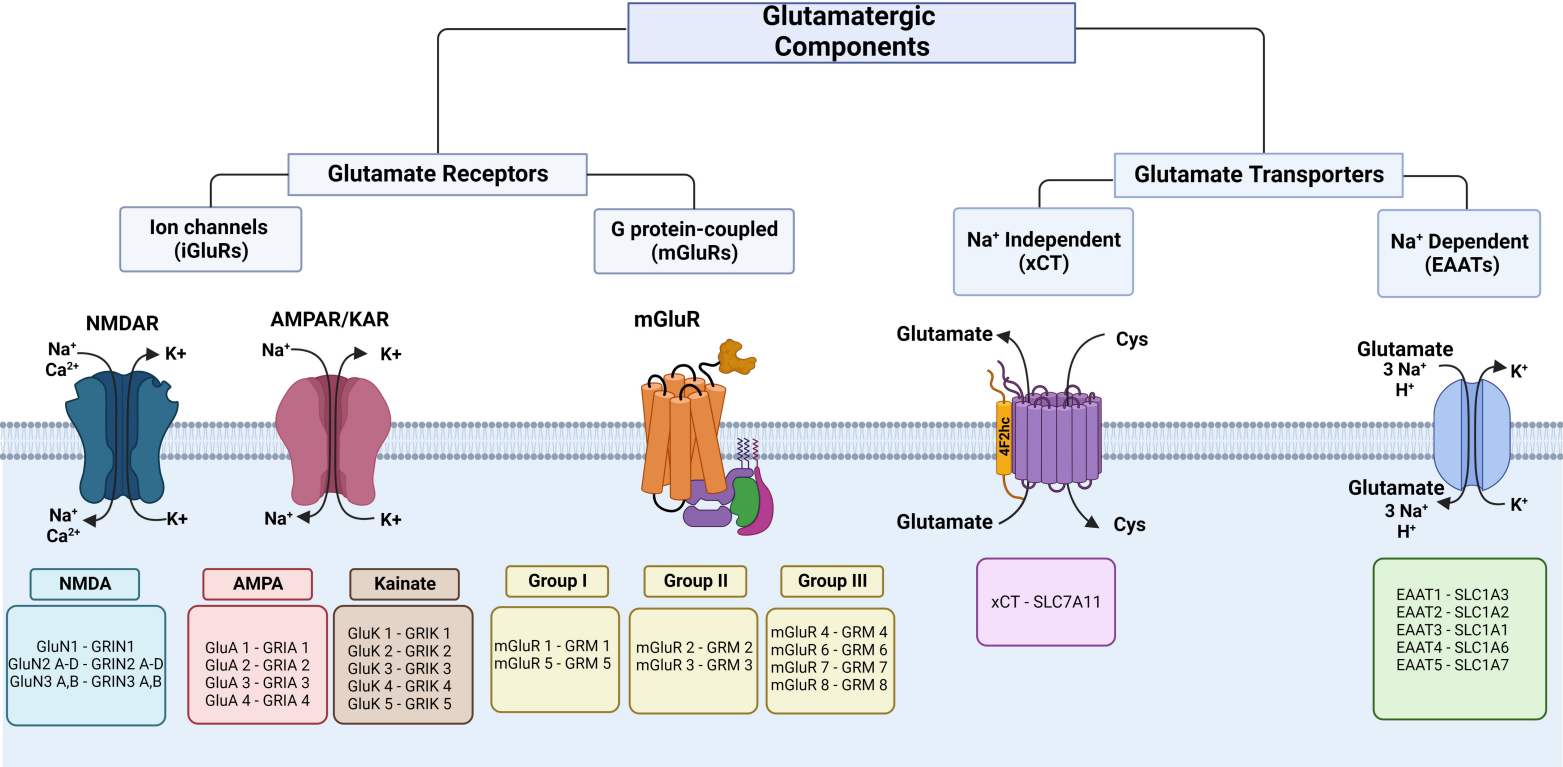
Classification of glutamate receptors and transporters. The left panel shows iGluR subunits and the respective coding genes. In the middle, the proteins and respective coding genes of mGluR families are shown. The right panel shows glutamate transporters of glutamate efflux and influx. Cys, cystine. This figure was created with BioRender.com.

iGluRs are glutamate-gated ion channels characterized based on the amino acid sequences, functional properties, and molecular structure, and are classified as NMDA (N-methyl-d-aspartate), AMPA (α-amino-3-hydroxyl-5-methyl-4-isoxazole-propionate), and kainate (**Figure 1**). iGluRs consist of a tetrameric or pentameric unit that forms an ion pore-forming segment (24, 25). In mammals, many different subunits form iGluRs: NMDA (GluN1, 2A-D, 3A-B encoded by Grin1, 2a-d, 3a-b), AMPA (GluA1-4 encoded by Gria1-4), and kainate (GluK1-5 encoded by Grik1-5) (to review the previous nomenclature, see the revision by Sprengel et al. (24)). These receptors are selective cation channels, allowing the passage of monovalent (Na^+^, K^+^) or divalent (Ca^2+^) cations (or both). iGluRs are regulated by postranscritional mechanisms like RNA splicing and editing, posttranslational modifications, like phosphorylation and ubiquitination, and trafficking to the plasma membrane (26, 27). In the CNS, these receptors have been widely characterized as mediators of synaptic transmission.

NMDA receptors mediate the majority of excitatory neurotransmission in the central nervous system (CNS) and Mg^2+^ blocks them at rested potential (28). After glutamate binding to NMDA receptors, rapidly depolarize the postsynaptic membrane and initiate signaling pathways (28). These receptors are highly permeable for Ca^2+^ and voltage dependent, showing high single-channel conductance (29). Under physiological conditions, cell membrane depolarization is necessary from a resting potential of -70 to -30–0 mV induced by the entrance of Na^+^ to release Mg^2+^ blocking and allow the activation of the channel by glutamate. In neurons, the released Mg^2+^ contributes to CREB activation by NMDA receptor in an extracellular calcium-independent manner (30). AMPA receptors support the depolarization that results in NMDA receptor activation. GluN1 is the main subunit that forms all functional NMDA receptors in the CNS (31). Glycine and D-serine serves as a co-agonist of glutamate for NMDA receptor activation (32, 33). The Ca^2+^ influx by brain NMDA receptors activates signaling *via* phosphorylation, activation of calmodulin-dependent kinase II (CaMKII) and protein kinase C (PCK) related to synaptic plasticity.

AMPA receptors evoke excitatory postsynaptic potentials and mediate the fast signal transmission of glutamate and receptor turnover in the CNS (27, 34). They are also involved in synaptic plasticity, which forms the basis of learning and memory, and display rapid desensitization (34). Posttranscriptional modifications make AMPA receptor subtypes that form homo and heterotetrameric channels highly diverse (35, 36) A specialized set of auxiliary proteins interacts with these receptors providing functional regulation (37). AMPA receptors are permeable to Na^+^ and K^+^ and a minor population is mainly present in interneurons for Na^+^ and Ca^2+^ (38, 39). Glutamate released by a presynaptic neuron activates AMPA receptors and depolarizes postsynaptic membranes that allow a pre-activated state of NMDA receptors to be activated by a second glutamate release.

Kainate receptors are formed by a tetrameric unit conformed by the low affinity for the ligand subunits GluK1-GluK3 and/or the high agonist affinity for the ligand subunits GluK4-GluK5 (26). These receptors are selective for Na^+^ and Ca^2+^ (24) and are regulated by postranscritional mechanisms like RNA splicing and editing, posttranslational modifications, and trafficking to the plasma membrane (26). Kainate receptors mediate slower synaptic transmission and are expressed both at pre-synapse, which occurs in excitatory neurotransmission, and at post-synapse which regulates neurotransmitter release. The iGluR proteins and coding genes are shown in **Figure 1**.

#### 3.1.2 Metabotropic glutamate receptors

mGluRs are seven transmembrane G-protein coupled receptors. In the CNS, they participate in increasing neuronal excitability and promote synaptic plasticity, which are key for learning and memory, through second messenger signaling pathways (40). These receptors are voltage-sensitive, and depolarization modifies the binding with G proteins and affinity for agonists (41, 42). mGluRs have been characterized into eight subtypes (mGluR1-8) encoded by GRM1-8 and classified into three groups (**Figure 1**) according to their pharmacology, sequence of amino acid homology, and signal transduction pathways (43). Group I (mGluR1 and mGluR5) is mostly localized post-synaptically, is normally stimulatory, and particularly, the subcellular location of mGluR1 in perisynaptic and extrasynaptic areas is associated with postsynaptic specialization of excitatory synapses (44), group II (mGluR2 and mGluR3) is found on post-synaptic and pre-synaptic cells and has a key function in reducing neurotransmitter release (45), and group III (mGluR4, mGluR6-8) is localized pre-synaptically and controls synaptic transmission (44). A variety of mGluRs that are expressed in different cell types play important roles in the immunology of the CNS, both in physiology and pathological processes associated with neurodegenerative disorders (44, 45). These receptors form homodimers, although they can eventually form heterodimers with other kinds of receptors, such as dopamine and adenosine receptors (46). Glutamate acts on mGluRs by activating gene expression and protein synthesis to exert a variety of regulatory effects through the recruitment of second messengers (47). Group I is coupled to Gq and activates phospholipase C, which produces inositol triphosphate and releases Ca^2+^ from intracellular stores in the endoplasmic reticulum, and diacylglycerol activates protein kinase C (47). Also, these receptors activate K^+^ channels and inhibit voltage-gated Ca^2+^ channels (48–50). Group II and group III are coupled to Gi and inhibit adenylate cyclase, and group III activates mitogen-activated protein kinases (MAPK) and PI-3 kinase (44, 47, 51). The mGluR proteins and coding genes are shown in **Figure 1**.

### 3.2 Glutamate receptors in cancer of non-neural organs

**Figure 2:**
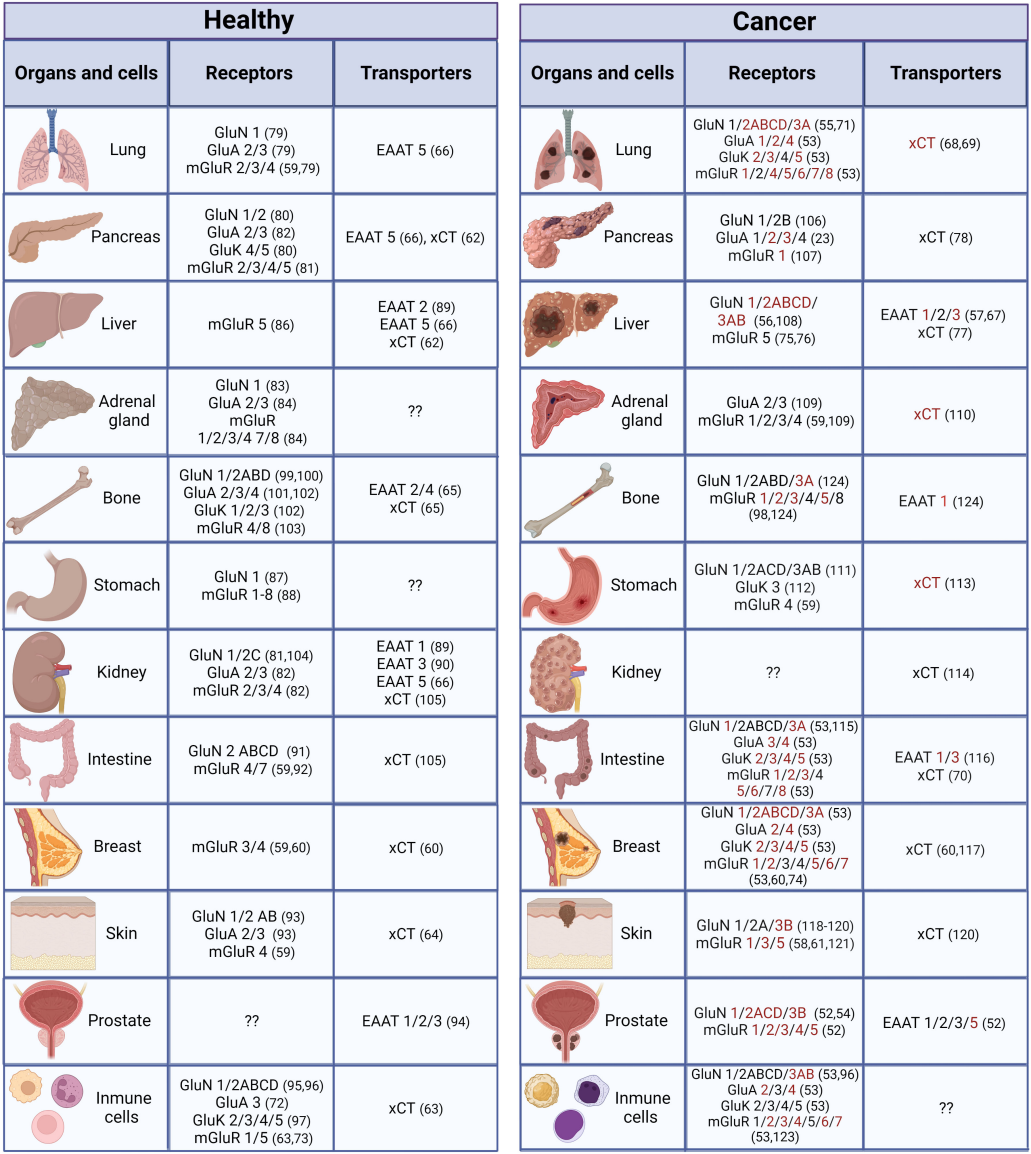
Distribution of glutamatergic system components in non-neural organs and cells under normal and cancer conditions. Components in red have only been reported under cancer conditions (they could be expressed *de novo* under cancer conditions or have not been studied under normal conditions). (23, 52–124).

Several glutamate receptors are expressed in a variety of organs and cells outside the CNS (**Figure 2**). Expression of both iGluRs and mGluRs was reported in androgen-dependent and androgen-independent prostate cancer cell lines (52) in human skin fibroblasts and various human cancer non-neural cells lines; the expression level of these receptors was lower in these cell lines than in the human brain (53). Upregulation, and in some cases downregulation, of glutamate receptors has been associated with cancer. Therefore, gain or loss of function could be possible for tumor survival with an aggressive environment associated with oxidative stress and inflammatory conditions. In recent years, studies have demonstrated that glutamate receptors are expressed in various types of cancer in non-neural cells, and although their precise involvement is still uncertain, there is strong evidence that they participate in different aspects of cancer development, such as augmented proliferation and metastasis, and adaptive metabolic changes (125, 126).

Regarding iGluRs, both NMDAR and AMPA have been detected by immunostaining in colon adenocarcinoma, lung, and breast carcinoma cell lines and it was reported that they favor proliferation in a Ca^2+^ influx-dependent manner (127). In particular, NMDAR1 expression was found in tissue sections of prostate, breast, and colon cancers, and has been proven to be functional in tumor-derived cell lines stimulating cell proliferation (54). NMDAR1 staining was observed in 145 prostate cancer specimens: positive stromal staining was low in 30%, moderate in 26%, and high in 15%, and either stromal or epithelial staining was observed in 81% of them. Of 18 benign prostatic hyperplasia specimens, 12 were normal prostate specimens, and none had stromal or scarce epithelial NMDAR1 staining (54). NMDAR1 and NMDAR2 have been identified in diverse cancer cell lines derived from peripheral sites, such as human lung adenocarcinoma cells A549, human breast cancer cells MDA-MB-231, and rat prostate cancer cells MAT-LyLu, and their activation also stimulated proliferation (128). In skin, functional NMDAR1 has been found in normal keratinocytes, especially at areas of cell-to-cell contact, influencing proliferation, differentiation, and migration during epithelialization, and playing a role in contact-mediated inhibition of growth. In squamous cell carcinomas, NMDAR1 is missing, which is consistent with the lack of contact-mediated growing (129). Conversely, overexpression of NMDAR1 has been associated with metastasis, tumor size, cancer stage, and poor prognosis in oral squamous cell carcinoma (130). This inconsistency can be explained because in this last study, authors found positive NMDAR1 reactivity in 50 of 81 cases from a matched normal adjacent mucosa, and the level of NMDAR1 was significantly associated with prognosis-related factors. Otherwise, researchers identified NMDAR2B expression in gastric epithelial cells and decreased or absent expression in a diversity of human cancer gastric cell lines (131). Accordingly, transcriptional silencing of NMDAR2B due to gene hypermethylation was identified in human cancer gastric cells, as well as in a variety of esophageal squamous carcinoma cell lines (131, 132). In this context, NMDAR2B emerges as a tumor suppressor gene since transfection of NMDAR1-1a and NMDAR2B significantly decreased viability and induced cell death by apoptosis of esophageal squamous cell carcinoma cell line KYSE140 in the presence of NMDA (132). NMDAR2B transcriptional silencing by methylation was also found in non-small cell lung cancer, which correlates with some clinical features (55), whereas NMDAR1 and 2A-D expression was observed in hepatocellular carcinoma tissue from rat and mouse models (56). Interestingly, in this model NMDAR1 and NMDAR2B expression was identified since fibrosis, a precancerous stage of liver disease, correlated with demethylation in the promoter regions (56).

Hypoxia is a common feature of the microenvironment of solid tumors that induces epithelial-mesenchymal transition and angiogenesis resulting in cell migration and metastasis. A hypoxic environment enhances AMPA receptor expression and signaling. In hepatocarcinoma cells, but not in breast cancer cells, GRIA2 and GRIA3 (GluA 2 and 3) mRNA expression is upregulated by hypoxia through HIF-1α and HIF-2α, which contribute to cell proliferation and growth of tumor xenografts (57). AMPA receptors have been demonstrated to promote proliferation and invasion in pancreatic cancer cells. GluA1 subunit levels were increased in pancreatic cancer precursor lesions, whereas GluA1, 2, and 4 subunit expression was scarce in pancreatic ductal adenocarcinoma accompanied by increased glutamate levels (23). Interestingly, in ductal carcinoma cells SU.86.86, activation of GluA receptors by a selective agonist induced invasion and migration, and inhibition of GluA by a selective antagonist decreased these parameters that were associated with oncogenic signaling *via* MAPK and K-Ras, as well as with changes in the presence of matrix metalloproteinase-2 (23).

An inflammatory environment could lead to the dysregulation of glutamate receptors, altering functions such as learning, spatial memory discriminatory or other information acquisition processes in the CNS (133). In this context, NF-κB is a proinflammatory transcription factor involved in carcinogenesis (134, 135). Some NMDA and AMPA receptor promoters contain NF-κB binding sites and are therefore sensitive to an inflammatory environment that might affect the gene transcription and/or remodeling of chromatin through epigenetic modification as has been observed in neural cells (136).

Regarding mGluR expression outside the CNS, numerous studies have demonstrated the presence of these receptors in the periphery. Twenty-five years ago, functional mGluRs in non-neural cells were found in rat hepatocytes in culture (137). Since then, studies have consistently found that glutamatergic receptors are involved in the development of inflammatory-related pathologies, especially cancer. A melanoma mouse model revealed the presence of mGluRs in the cancer biology (138). Additionally, in human melanoma biopsies and cell lines, but not in benign tissues and melanocytes, expression of GRM1 (mGluR1) was detected (138). Mutations in mGluR3 have also been identified in melanoma, which were associated with increased anchorage-independent growth and migration (58). mGluR overexpression is associated with pathological clinical parameters and poor disease-free survival in cancer. For example, mGluR4 overexpression is related to a poor prognosis in colorectal carcinoma (59). Expression of mGluR4 was detected in 54% of 241 samples of colorectal carcinoma and, interestingly, 5% of them showed cytoplasmic expression; the association with clinical features was positive with recurrence and poor disease-free survival. Additionally, mGluR4 overexpression seems to mediate 5-fluorouracil resistance in colon cancer cells and its activation by the agonist L-AP 4 promotes cell survival (139). It seems that the presence of mGluR4 is detrimental for patients with colon cancer. mGluR3 is upregulated in most human colonic adenocarcinomas and colon cancer cell lines. Its reduction by knockdown expression decreased cell survival *in vitro*, whereas *in vivo*, it inhibited tumor growth by enhancing TGFβ tumor suppressor function (140). In breast cancer, mGluR3 promotes invasiveness through activation by autocrine action of glutamate generated by glutaminolysis exported to the extracellular milieu *via* the xCT transporter (60). In our hands, overexpression of mGluR3 was observed in the progression of cirrhosis to hepatocarcinoma development in rats and in HepG2 cell line, suggesting a role in the pathophysiology of the liver cancer (A.C. García-Gaytán, A. Hernández-Abrego, D. De Ita-Pérez, E. de los Ríos-Arellano, I. Turrubiate, E. Gámez, M. Díaz-Muñoz, I. Méndez, unpublished data).

mGluR1 can be an oncogene in epithelial cells and critical in the genesis of melanoma. Transfection of GRM1 induces transformation of mouse kidney cells that exhibit enhanced cell proliferation *in vitro* and significant tumorigenicity *in vivo* involving MAPK and AKT signaling, both crucial for tumorigenesis (141). High expression of mGluR1 was observed in primary or metastatic prostate cancer tissues, while in luminal acinar epithelial cells of normal or hyperplastic glands the expression was weak or absent (142). Moreover, mGluR1 is expressed with high prevalence in most breast cancer subgroups, such as invasive breast cancer with positivity in 60% of the ductal tumors and 47% in lobular tumors and is an unfavorable prognostic marker (143, 144). mGluR1 expression was associated with a lower tumor grade and with estrogen receptor-positive and progesterone receptor-positive tumors, suggesting that hormone stimulation regulates mGluR1 expression (144). Analysis of the progression from normal epithelium to triple-negative breast cancer demonstrated that mGluR1 overexpression induces transformation to a malignant phenotype that was reverted by silencing the GRM1 gene (143). Moreover, the expression of mGluR1 lead to the transformation of hyperplasic triple-negative breast cancer cells in human breast cancer cell xenografts in athymic nude mice (143). In addition to overexpression, mutations and single nucleotide polymorphisms or SNPs of GRM1 have been identified in multiple cancers, such as prostate (145), colorectal, and lung (146), that led to alterations in receptor activation, intracellular localization, and downstream signaling. mGluR5 expression induced melanoma in transgenic mice under a melanocyte-specific promoter, showing an aggressive phenotype with early onset (61). Hence, some of the mGluRs could be considered as prognostic markers and possible therapeutic targets for certain types of cancer.

### 3.3 Glutamate transporters

As the major mediator of excitatory signals in the nervous system of mammals (147), tight regulation of extracellular glutamate concentrations at both synaptic and extra-synaptic locations is critical to ensure correct neurotransmission and prevent neurotoxicity events. In the CNS, glutamate is stored, and subsequently released from the presynaptic terminal to neighboring neurons by exocytosis, with successful replenishment by specialized recycling synaptic vesicles known as vesicular glutamate transporters (VGLUTs) (148). In non-neural organs, expression of some VGLUTs has been found in intestinal cells, pancreas, and osteoclasts; these VGLUTs contribute to glutamate release and exert autocrine and paracrine actions (149–151). VGLUT expression in liver, stomach, testis, and platelets suggests that they have numerous functions in the physiology of these sites (152–154). There is evidence of VGLUT expression in carcinoma stem cells that correlates with enhanced release of glutamate and cell proliferation *via* activation of NMDA receptors (155); therefore, VGLUT functions could also be implicated in cancer development.

In non-neural tissues, plasma membrane transporters act as non-vesicular routes of glutamate release or uptake. Non-vesicular releasing is the main source of extracellular glutamate in the brain, such as gap junction hemichannels (156) and heteromeric sodium-independent amino acid transporters, particularly system xCT (157).

#### 3.3.1 System xCT antiporter

**Figure 3:**
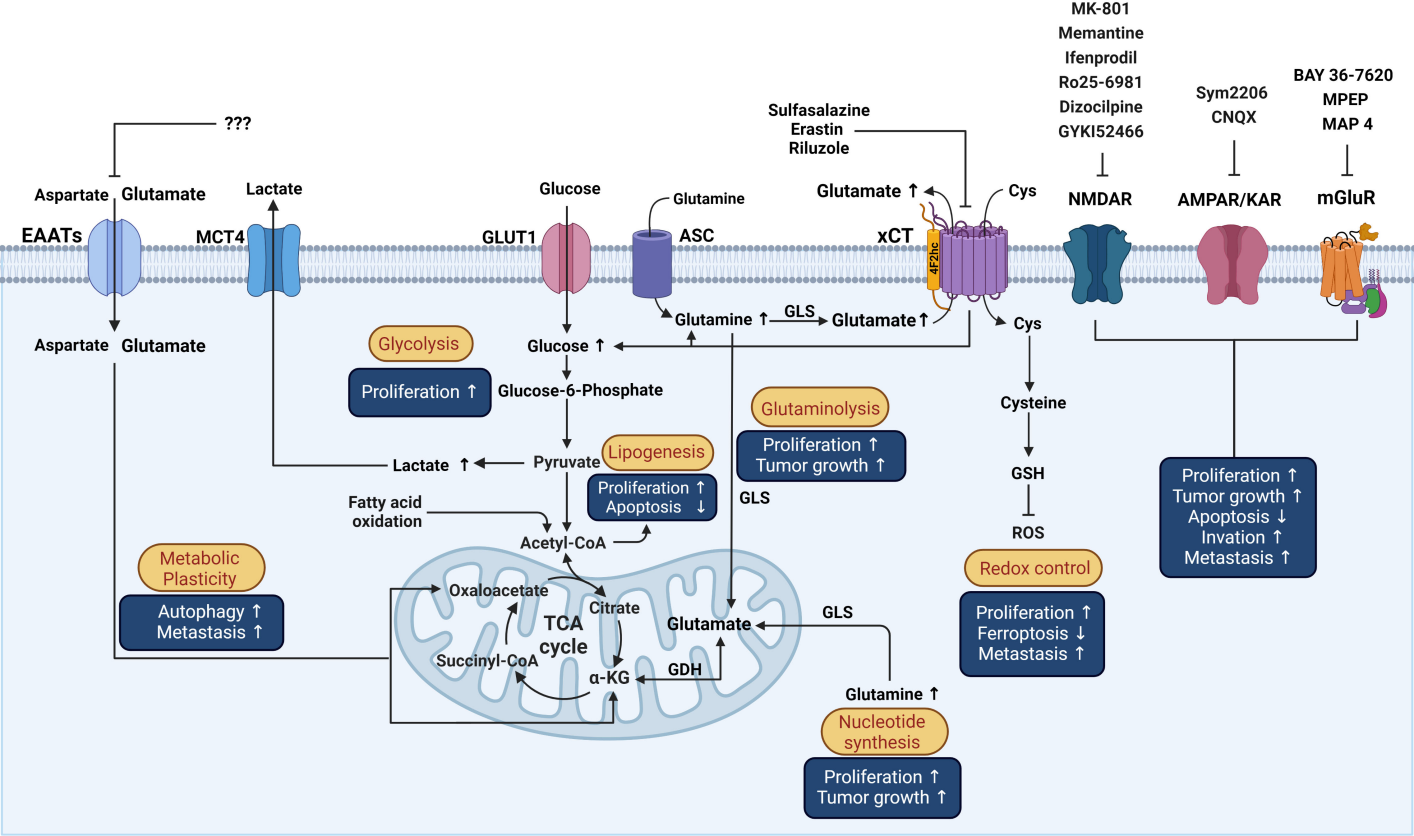
Schematic representation of the influence of glutamatergic system components on reprogrammed metabolism in cancer cells and the effects over processes associated with cancer. Possible actions of known drugs that affect glutamatergic components over different mechanisms involved in the pathophysiology of cancer that are described in the text are shown. Cys, cystine; GSH, Glutathione; GLS, Glutaminase; ASC, Alanine/serine/cysteine transporter; GDH, Glutamate dehydrogenase; α-KG, alpha-ketoglutarate; ROS, reactive oxygen species; TCA, Tricarboxylic acid cycle; GLUT1, Glucose transporter 1; MCT4, Monocarboxylate transporter 4. This figure was created with BioRender.com.

System xCT (Sx_c_^-^) is a cystine/glutamate Na^+^-dependent antiporter that mediates the exchange of extracellular L-cystine and intracellular L-glutamate at a 1:1 ratio across the cellular plasma membrane, that is involved in neural protection against cerebral degeneration or damage (158–160). This system is a member of the heteromeric amino acid transporter family (glycoprotein‐associated amino acid exchangers) consisting of a heterodimer formed by the transporter subunit or light chain xCT (SLC7A11) and a regulatory subunit or heavy chain 4F2hc (4F2 cell-surface antigen heavy chain, acronyms: CD98, rBAT, FRP1, slc3 family SLC3A2) covalently linked through a disulfide bond to xCT (161). The xCT subunit is a hydrophobic, non‐glycosylated protein that has transport activity (161). 4F2hc acts as a chaperone for several amino acid transporters which serves to regulate trafficking to the plasma membrane maintaining xCT protein stability. xCT activity supports the non-vesicular release of glutamate and is critical for oxidative protection through reduced glutathione (GSH) production and provides intracellular L-cystine that is rapidly reduced in the cytoplasm to L-cysteine, a precursor for the enzymatic synthesis of GSH (162) (**Figure 3**).

xCT is present in neurons and glia, especially in microglia and astrocytes, in the CNS, is the largest source of extracellular glutamate in the brain, and is dysregulated in neurological conditions (163, 164) and in various organs and cell types outside the CNS, such as liver, pancreas, skin, bone, dendritic cells, mammary epithelial cells, and fibroblasts, among others (60, 62–65, 158) (**Figure 2**). Interestingly, in the cancer stage, xCT is upregulated in some of those sites (e.g., breast cancer cells) (60) or expressed *de novo* (**Figure 2**). Several mechanisms are involved in the upregulation of xCT expression by amino acid deprivation, xenobiotics and, particularly, oxidative stress (165, 166). The production of reactive oxygen species (ROS) provokes cellular responses such as transcriptional activation of genes encoding proteins that participate in the mechanisms of protection against oxidative stress by xCT. Antioxidant response element (ARE) *cis*-acting sequences are present in the promotor regions of xCT that responds to phosphorylated nuclear factor erythroid‐2‐related factor (Nrf2) and increases its transcription (165, 167). Likewise, a proinflammatory environment increases the efflux of L-glutamate and induces the increase in L-cystine uptake through xCT in neurons co-cultured with astrocytes, leading to hypoxic neuronal injury (168). Both oxidative stress and inflammation promote oncogenesis. Sustained oxidative stress can induce chronic inflammation through the activation of several transcription factors, including NF-κB, p53, HIF-1α, and Nrf2, involved in the distinctive characteristics of cancer such as cell proliferation, migration, and survival (169).

Extracellular glutamate is not metabolized by any enzyme (170). Therefore, glutamate is able to interact with receptors until it is removed into the cell by specialized transporters located on the plasmatic membrane of pre- and post-synaptic neurons and astrocytes that play an essential role in the control of extracellular glutamate concentrations, with a high level of cooperation. Outside the CNS, glutamate reuptake is carried out by transporters that mobilize glutamate from the extracellular space.

#### 3.3.2 Excitatory amino acid transporters

Glutamate uptake is accomplished in the CNS by excitatory amino acid transporters (EAATs) that are present in the pre- and postsynaptic cell surface of neurons and astrocytes, where glutamate is preferentially recycled (163). EAATs belong to the solute carrier (slc) family 1 and transport L-glutamate and DL-aspartate with similar affinity. Five human EAATs have been identified: EAAT1-5: glutamate−aspartate transporter (GLAST/EAAT1), glutamate transporter (GLT/EAAT2), excitatory amino acid carrier (EAAC/EAAT3), and excitatory amino acid transporters 4 and 5 (EAAT 4 and EAAT5) (163) (**Figure 1**). EAAT1-3 are high-capacity glutamate transporters that play an important role in the regulation of the vulnerability to excitotoxicity modulating accumulation of glutamate, its receptors activation, and its metabolic homeostasis (171, 172). In addition, EAATs also serve as anion channels. EAATs catalyze coupled transport of 1H^+^ and 3Na^+^ with one molecule of glutamate and counter-transport 1K^+^ (**Figure 1**) (147, 173). Some EAATs operate as chloride channels in response to the glutamate transporter function (174). EAATs assemble as homo- or heterotrimers; each subunit exerts transport actions independently of the others (175) and form subpopulations of transporters with different affinities for glutamate (162). Their expression is under different mechanisms of regulation including transcriptional, epigenetic, and posttranslational control, that may influence on the trafficking and availability in the plasma membrane (162). Altered expression has been observed in pathologies such as Alzheimer disease that could indicate alterations in glutamate recycling (176). The mTOR signaling pathway is often activated in cancer (177) and is a potent regulator of EAATs (162). In peripheral organs, various EAATs express at the mRNA and protein levels in diverse sites, such as liver, kidney, and pancreas, among others (**Figure 2**) (66, 67).

### 3.4 Glutamate transporters in cancer of non-neural organs

During tumorigenesis, metabolic flexibility comprises reprogramming of catabolic and anabolic pathways to contend with their demands for energy, growth, proliferation, and detoxification, which involve the handling of several substrates related to aberrant glucose, lipid, and glutathione-glutamine-glutamate metabolism (178, 179). Metabolic reprogramming in cancer also increases oxidative stress; therefore, cancer cells tend to upregulate their antioxidant defenses in the form of GSH to maintain the appropriate redox balance. System xCT is instrumental in metabolic reprogramming, as it increases glucose consumption and lactate production, as well as glutamine dependency of cancer cells (68) (**Figure 3**). The oncogene RAS influences the transactivation of xCT promoter, enhancing GSH synthesis as part of the protection mechanism to favor oncogenic transformation (69). Thus, system xCT overexpression frequently found in an array of malignant tumors promotes survival features at the expense of increased GSH synthesis, such as protection from lipid peroxidation and oxidative stress-induced cell death (180, 181), cell proliferation, invasion, tumor growth, and chemoresistance (68, 70, 182–184). Conversely, there are cancer cells with a low expression level of xCT, such as chronic lymphocytic leukemia, which uses the capacity of stromal cells to synthesize GSH for survival and drug resistance to phenethyl isothiocyanate (185).

In contrast to the well-established pro-survival effects of xCT overexpression, xCT in renal cancer cells enhances cancer dependence on glucose and renders cancer cells more sensitive to cell death induced by glucose starvation (186). In fact, glucose starvation induces xCT overexpression through its major inductor Nrf2 transcription factor. It appears that xCT overexpression in these conditions serves as a ROS scavenger and protects the cells from glucose starvation. The bimodal role of xCT in protecting and promoting cell death in cancer make it challenging to elaborate therapeutic strategies that alleviate the symptoms caused by cancer. In this regard, it is important to consider the context of the disease, namely the oncogenic nature of the cancerous cell.

Regarding the role of the EAATs in the physiopathology of cancer, a differential expression of the EAAT family members has been observed in human cancers (187). An aberrant expression of several members of this family correlates with immune infiltration, tumor grade, and cancer stage, and nodal metastasis status in lung adenocarcinoma has a promising prognostic value and is a potential target for therapy in this cancer (187). In an analysis from The Cancer Genome Atlas, lung cancer patients with high expression of EAAT3 exhibited poor survival rate compared to patients with low EAAT3 expression, thus supporting the notion that EAAT3 is important in lung tumor progression (188). Overexpression of EAAT1, EAAT2, and EAAT3 contributes to cancer progression in non-neural solid tumors, such as gastric and lung cancers (188–190). EAAT1 and EAAT2 have been identified in adenocarcinomas from intestine, stomach, and genital tract and, depending on the cell confluence related to touching among cells, they re-locate from the plasma membrane to the nucleus, such that they no longer promote glutamate clearance when the cells are not in touch (191). While normal cells preserve membrane localization of EAATs, their mislocalization in malignant cells together with up-regulation of xCT lead to aberrant handling of glutamate that induces high levels of glutamate in the extracellular space, as seen in gliomas (192) and in breast cancer, where endocrine therapy-resistant cells promote autophagy and glutamate import *via* EAAT2 (60, 193). Interestingly, the relocation of EAAT1 and EAAT2 does not occur in astrocytoma; therefore, the regulation and effects of glutamate transporters should not necessarily be the same in all tumor types.

Several cancers are treated efficiently with endocrine therapy. However, it has been observed that a significant group of patients with breast cancer relapsed with the endocrine therapy-resistant disease. This pattern is associated with an enhanced influx of aspartate and glutamate *via* EAAT2 that serves as fuel for metabolic pathways and promotes autophagy and metastasis (193). Moreover, higher expression of EAAT2 is linked to changes in proliferation induced by treatments with an antagonist of estrogen receptors (fulvestrant) or an aromatase inhibitor (letrozole). Hence, the increased activity of EAAT2 promotes resistance to therapy that reinforces the overexpression of EAAT2 in a feedback loop. The challenge would be to end the vicious circle as a therapeutic strategy. Altogether these findings support a role for glutamate transporter EAATs in therapy-resistant cancer.

Hypoxia inducible factors (HIFs) mediate the transcription of multiple genes that allow cells to adapt to hypoxic environments, increase in cancer with a poor prognosis, and participate in various stages of carcinogenesis and cancer progression (194). In cells derived from hepatocarcinoma and renal carcinoma, but not from breast cancer, EAAT1 and EAAT3 expression is upregulated under hypoxic conditions through transactivation of gene expression by HIF-1α and HIF-2α (57). Hypoxia favors glutamate signaling through upregulation of AMPA receptors and EAATs. Since glutamate transport by EAATs could be reversible in the outward direction in neurons under certain conditions (e.g., when ratio [Na^+^]i/[K^+^]e decreases) (195), it seems that something similar occurs in hepatocarcinoma. This supposition needs further investigation.

Another member of the EAAT family, EAAT1, has been identified as a contributor to tumor initiation and progression, as well as asparaginase resistance in prostate cancer and breast cancer cells (196). Additionally, EAAT1 overexpression promotes cancer growth and correlates with a poor prognosis in a xenograft tumor growth model of gastric cancer (190). EAAT1 has been proposed as a potential biomarker for the pathogenesis and progression of chondrosarcoma (197).

Recent evidence suggests increasingly important actions of EAATs in various hallmarks of cancer development, including cell proliferation, reprogramming of energy metabolism, and drug resistance. Therefore, EAATs could be considered as another therapeutic target in searching for a more specific cancer treatment.

## 4 Glutamate system as a diagnostic tool and therapeutic targeting in non-neural organs

Progress in the management of cancer including chemotherapy, radiotherapy, and immunotherapy has been substantial (198). However, along with resistance to therapy, there is still the challenge of developing personalized therapy with fewer side effects. For many years, combined modality therapies have been developed for complete tumor eradication (198, 199). In this context, glutamate system components might be good candidates for concomitant therapy approaches with known treatments to avoid resistance, diminish toxicity, and offer individualized cancer treatment.

### 4.1 Glutamatergic receptors in diagnosis and therapy

Malignancy frequently induces pain sense in peripheral organs that are innervated by afferent fibers. Several organs such as colon and liver produce and release glutamate, which mediates the activation of locality-specific nociceptive neurons (200). Another source of glutamate might be peripheral innervation like that of the epidermis (201). Pharmacological approaches have proposed antagonists of mGluRs and NMDA receptors in afferents that innervate peripheral organs, such as skin (202), colon (203), and bone (204), as therapeutic targets for the treatment of pain, since they have obtained analgesic effects. There is evidence that glutamate levels might increase in the serum or plasma of patients with cancer (205, 206); thus, they have been suggested as a biomarker of diagnosis or prognosis. Significantly higher serum glutamate levels correlated with aggressiveness in prostate cancer patients (142). Also, elevated glutamate levels were found in the serum of patients with hepatocellular carcinoma compared to those of patients with cirrhosis and healthy controls (207). Higher glutamate concentrations in the extracellular space could bind and signal through receptors to produce biological effects on the physiopathology of non-neural cells, independent of nervous system fibers. In addition to the high levels of circulating glutamate, the expression of other components of the glutamatergic system could serve as biomarkers and/or therapeutic targets for improving currently approved treatments.

In patients with oral squamous cell carcinoma, NMDAR1 is a likely prognostic biomarker and therapeutic target for cancer, as NMDAR1-positive tumors are associated with poorer survival compared to NMDAR1-negative tumors (130). Differential expression of NMDAR1 has been detected in stromal and epithelial cancer prostate tissues while a lack or scarce expression was observed in normal or hyperplastic prostate specimens (54), suggesting that NMDAR1 expression could be a biomarker for prostate cancer. In small-cell lung cancer cell lines, NMDAR1 and NMDAR2 promoted cell proliferation, and positive immunostaining for NMDAR1 was found in human tumor samples (71). Treatment of these small-cell lung cancer cells with the NMDAR1 antagonists dizocilpine (MK-801) and memantine impaired cell viability, whereas treatment with the NMDAR2 antagonists ifenprodil and Ro25-6981inhibited cell growth (71). Memantine has been proven to inhibit the growth of human prostate cancer cell lines (54). Moreover, dizocilpine prevented proliferative actions induced by epidermal, insulin, and basic fibroblast growth factors by upregulating the tumor suppressor protein p21 in lung adenocarcinoma cells and prolonged the survival in mice that were injected with human lung carcinoma cells into the peritoneal cavity (128).

Studies have shown that using antagonists of distinct iGluRs is effective because they exert anti-proliferative actions, inhibit migration, and alter the morphology of tumor cells, and that they have synergistic effects when used with chemotherapy agents, such as cisplatin or cyclophosphamide (127). The effects depend on the type of cancer and iGluR. For example, in colon adenocarcinoma and breast and lung carcinoma, cells are highly sensitive to the NMDAR antagonist dizocilpine and to the GluA 2/3 antagonist GYKI52466 (127). Treatments with iGluR antagonists resulted in modified membrane protrusions and thus a decreased motility and invasive phenotype of the thyroid carcinoma cells FTC238 (127). In addition, oncogenic signaling *via* MAPK and K-Ras was reduced by the non-competitive AMPA receptor antagonist SYM2206, and the invasiveness and migration were inhibited in the ductal pancreatic cell line Su86.86 (23). These data support the assessment of drugs that target iGluRs for potential uses in anticancer therapy.

On the other hand, DNA methylation pattern has emerged as a promising molecular biomarker for cancer detection, prognosis, and therapeutic response (208). Hypermethylation in promoter regions of cancer-associated genes can lead to blocked transcription (209). Accordingly, hypermethylation of NMDR2B detected in primary human esophageal squamous cell carcinoma and in primary gastric cancer tissues was associated with suppression of tumor features such as proliferation and colony formation (131, 132). Restoration by NMDAR2B transfection and activation in esophageal squamous cell carcinoma induced the suppressor phenotype eliciting cell death by apoptosis (132). Otherwise, increased expression of NMDAR1 and 2B was identified in hepatocellular carcinoma. This expression appeared from a precancerous stage of liver disease correlating with demethylation in CpG islands in the promoter regions in rat and mouse models of hepatocellular carcinoma (56). NMDAR2B emerges as a tumor suppressor gene since transfection of NMDAR1-1a and NMDAR2B significantly decreases viability and induces cell death by apoptosis of esophageal squamous cell carcinoma cell line KYSE140 in the presence of NMDA (132). Hence, the expression level of NMDARs and the methylation or demethylation of NMDAR in promoter regions might provide biomarkers to assess therapy response in these types of cancer.

In the immune system, expression of a plethora of iGluRs and mGluRs in human T cells has been reported in resting and activated cells and under normal and cancer conditions (210, 211). Both types of glutamate receptors have been shown to contribute to diverse functions. For example, iGluRs expression in T cells induces adhesion and chemotaxis (72), mediated by Ca^2+^ signaling, whereas mGluR promotes survival protecting from T cell activation-induced cell death (73). Additional evidence supports that glutamate released by dendritic cells through xCT antiporter acts in a paracrine way on mGluR1 to activate T cells (63). Considering that cancer cells avoid the immune response, activation of T cells *via* glutamate receptors could provide another effective T cell-mediated immune response against the tumor. Additionally, iGluRs and mGluRs expressed in non-solid tumors, T-cell leukemia, and T-cell lymphoma promote cancerous T cells by augmenting their extravasation and the expression of matrix metalloproteinases and evoking iCa^2+^ increase and concomitant expression of genes that regulate the cell cycle, respectively (211). Blocking AMPA receptors with antagonists reverted the effect over increased secretion of matrix metalloproteinases and limited tumor growth, suggesting that glutamate receptor inactivation could be another possible therapeutic strategy against cancer (212).

Regarding mGluRs as therapeutic targets, some drugs have been suggested as possible treatments for solid cancers. mGluR1 is visualized as a prognostic marker and a potential treatment target in some types of cancer. In triple-negative breast cancer cells, both a selective mGluR1 antagonist and silencing with shRNA targeting the gene GRM1 inhibited proliferation associated with apoptosis (143, 213). Interestingly, MDA-MB-231 triple-negative breast cancer cells and normal AB589 mammary epithelial cells express GRM1; however, treatment of cells with the mGluR1 antagonist BAY 36-7620 had a significant effect on cell growth only in breast cancer cells (213). *In vivo* research using MDA-MB-231 xenograft-bearing mice found that both the mGluR1 antagonist and riluzole were effective in reducing tumor volume (213). Riluzole is an oral drug with low toxicity that could be a therapeutic candidate for cancer therapy. This drug has been shown to inhibit the proliferation of human melanoma cell lines that express mGluR1 (22). Moreover, treatment of human melanoma cell xenografts in immunodeficient nude mice with riluzole inhibited tumor growth by 50% (22). A phase II trial of riluzole in melanoma seems to be promising (214).

mGluR1 has been suggested as a pro-angiogenic factor and a mediator of tumor progression in breast cancer (74). In this regard, primary endothelial cells and endothelial cell lines were shown to express high levels of mGluR1, and both riluzole and the non-competitive antagonist BAY36-7620 significantly inhibited cell proliferation *in vitro* and angiogenesis in subcutaneous implants *in vivo* (74). These data suggest that the inactivation of mGluR1 may represent a promising therapy for cancer related to angiogenesis. Although there was varying levels of mGluR5 in human oral squamous cell carcinoma tissues from patients, a significant positive association with overall survival was found (215). Moreover, the mGluR5 activation with the agonist DHPG increased tumor cell migration, invasion, and adhesion in the HSC3cell line, effects that were reverted by the antagonist MPEP (215). Inactivation of mGluR5 with the selective antagonist MPEP suppressed oncogenic actions; for instance, it induced inhibition of cell growth, migration, and invasion *via* ERK phosphorylation and induced apoptosis in the cell line HepG2. It also inhibited metalloproteinases 2 and 9, as well as tumor growth and metastasis in a xenograft model of hepatocellular carcinoma (75). mGluR5 expression is higher in hepatocellular carcinoma and chemotherapy agents caused cell death by decreasing mGluR5 in this cancerous system and increasing mGluR5 in normal hepatic cells. Moreover, inhibition of mGluR5 was shown to enhance the potency of chemotherapeutic agents in hepatocellular carcinoma cell lines and attenuate chemotoxicity over DNA damage by downregulating Ca^2+^-dependent MAPK signaling in the normal liver (76). Hence, mGluR5 seems to be a prognostic marker and a possible therapeutic target in oral squamous cell carcinoma and hepatocarcinoma.

Therefore, the pharmacology of glutamate receptor activity may be therapeutically useful for cancer treatment.

### 4.2 Glutamate transporters in diagnosis and therapy

Overexpression of xCT correlates with tumor invasion, short survival, and a poor prognosis in patients and animal models of hepatocarcinoma (77, 216), acute myeloid leukemia (217), non-small cell lung cancer (68), prostate cancer (218), and colorectal cancer (219). Therefore, xCT has been considered as a possible biomarker of cancer progression and recurrence, and also as a therapeutic target for various types of cancer (77, 220). Moreover, xCT overexpression renders cancer cells, such as ovarian cancer cells, tongue squamous cell carcinoma cells, lung cancer cells, and gastric cancer cells, more resistant to chemotherapy with cisplatin (221–224).

Chemoresistance is still a challenge in cancer therapy. Chemotherapy-resistant cancer cells rely on cystine import for ROS resistance *via* the xCT. In addition to being a biomarker, xCT has been proposed as a therapeutic target for drug-resistant cancer types, such as triple-negative breast cancer, pancreatic cancer, and hepatocarcinoma, that depend on xCT for survival (78, 220, 225, 226). Patients with hepatocellular carcinoma with high xCT mRNA expression showed poorer overall and disease-free survival than those who lacked xCT mRNA (77). Therefore, xCT could be used as a biomarker for disease prognosis.

Multiple studies have demonstrated that cancer stem cells can promote tumorigenesis, tumor growth, and chemoresistance (227–229). Cluster of differentiation 44 (CD44) is an adhesion molecule in cancer stem cells that does not seem to be involved in stem cell properties and interacts with xCT. CD44 upregulates and stabilizes the membrane insertion and activity of xCT by interacting with the complex xCT-4F2hc (224, 230) to regulate the intracellular level of GSH, thereby controlling the defense against ROS generation. In gastric cancer, ablation of a CD44 variant (CD44v) diminishes membrane xCT and suppresses tumor growth accompanied by a greater expression of phospho-p38 MAPK and upregulation of p21 in the cancer cells that adopt a more differentiated and less proliferative state (230).

It has been demonstrated that xCT deficiency or inhibition suppresses cell proliferation and tumor growth and induces cell death by apoptosis or cell death mediated by accumulation of lipid ROS or lipid peroxidation, known as ferroptosis (231–234). Moreover, xCT inhibition potentiates the cytotoxic effects of gemcitabine and cisplatin in pancreatic ductal adenocarcinoma cells (234).

Inhibitors of xCT have been observed with therapeutic potential for the suppression of tumors. Sulfasalazine, a specific inhibitor of xCT and FDA-approved drug, is a potent suppressor of tumors that inhibits growth, invasion, and metastasis as has been revealed by the attenuation or suppression of lymphoma growth (231), metastasis of melanocytes (235), proliferation, colony formation, metastasis and invasion of gastric cancer cells (236), and stemness and metastasis of colorectal cancer (237). Further, sulfasalazine overcomes ionizing radiation and chemoresistance to anti-cancer therapies in hepatocarcinoma (183), improves cisplatin chemosensitivity in cholangiocarcinoma (238), and enhances the sensitivity to cell death by ferroptosis in paclitaxel-resistant uterine serous carcinoma cells (239). Repeated cisplatin treatment-induced cisplatin-resistant cells present high expression of xCT. Using different strategies, such as xCT inhibitors (sulfasalazine or erastin) or xCT siRNA, could sensitize human gastric cancer cells to cisplatin (240). Likewise, sulfasalazine suppresses CD44-dependent tumor growth promoting p38 in gastric cancer cells (230). In fact, hepatocarcinoma treated with hepatic arterial infusion chemoembolization therapy with cisplatin expresses a high level of CD44v9, suggesting that it is involved in the resistance to treatment (241). Interestingly, treatment with sulfasalazine was effective in combination with cisplatin in the hepatocarcinoma CD44v9-positive cell line HAK-1A (241). Sulfasalazine has been shown as a good candidate for anti-cancer therapy; however, its specificity is limited since its effects depend not only on the type of cancer but on other features of cancer cells of the same type of cancer. For example, in lung cancer, inhibition of proliferation in the cell lines A549 and H520 in response to sulfasalazine was not observed in the H1869 cell line (68). Inhibition of xCT alone is not sufficient since cysteine is still imported by additional transporters as the ASC system; thus, enzymatic treatment to eliminate extracellular cysteine has been proposed for prostate cancer treatment (242). Accordingly, it is still necessary to investigate the extent to which sulfasalazine, sorafenib, erastin analogs or any other xCT inhibitor could be harnessed for cancer therapy (70, 243). Erastin and sorafenib are antitumor agents that induce ferroptosis cell death by inhibiting xCT (244, 245). Even though their benefits as monotherapy are limited in survival because of primary and acquired resistance (246–248), they are able to enhance the sensitivity of chemotherapy and radiotherapy and function significantly better together with another antineoplastic agent (244, 249). Cancer immunotherapy is based on antigen-specific T cell function and is a potent toll against cancer with the advantage that it is better tolerated than common chemotherapy drugs (250). Immunotherapy functions well by itself for some types of cancers, and for others it works better together with other treatments. Combined treatment using immunotherapy results in a significant increase in durable responses, which suggest a viable strategy for improving anticancer immunotherapy (184). There is evidence that xCT deficiency does not impair T cell proliferation and antitumor immunity *in vivo* (184). Since xCT inhibition is dispensable for T cell proliferation, combined therapy of xCT inhibition with immunotherapy is a promising therapeutic strategy for cancer. Another strategy using vaccination of anti-xCT has been tested with good results on improving sensitivity to chemotherapy by doxorubicin in tumor growth and metastasis of breast cancer (251). Therefore, xCT-targeted therapy would impede ROS defense and compromise cell survival and may be ancillary, or even synergic, for other conventional treatments, such as chemotherapy or immunotherapy in refractory cancer cells but may be more susceptible to xCT-target drugs (252).

Glutamate transporters influence each other since the overexpression of some affects others. In non-small cell lung cancer, EAAT3 overexpression facilitates recycled extracellular glutamate into the cells by enhancing xCT activity and the consequent GSH biosynthesis to favor tumor growth and a malignant phenotype (188). Thus, increased extracellular glutamate by EAAT3 inhibition would restrict cystine uptake by xCT and promote cell death as a result of the blocking of GSH synthesis.

Regarding the role of EAATs in cancer therapy, it has been shown that the EAATs mediate therapeutic resistance related to altered tumor metabolic profiles. Cancer treatment schemes with enzymes that catabolize amino acids to deplete them have been successfully used in solid tumors with auxotrophy for arginine or asparagine with high clinical efficacy (253, 254). The limitation of this treatment is the intolerant toxicity (255) and therapy resistance, because cancer cells remain proliferative despite asparaginase treatment, the mechanism of which little is known. In this regard, SLC1A3 (EAAT1) has been identified to contribute to asparaginase resistance in prostate and breast cancer in which combined treatment with asparaginase and EAAT1 chemical inhibition restrains cancer cell proliferation and tumor progression (196). In response to this combination, metabolism is altered in prostate and breast cancer cells, including NAD^+^/NADH inhibition and lactate depletion which are important indicators of energy and redox status. It is clear that EAAT1 participates in enzyme treatment resistance. On the other hand, an RNA sequence analysis showed that SLC1A6 (EAAT4) was upregulated in nasopharyngeal carcinoma cell lines resistant to radiation and with low sensitivity to cisplatin (256). In addition, radiation upregulates EAAT4 expression in radioresistant cell lines that correlates with poor prognosis in patients with nasopharyngeal carcinoma (256).

In the complex context of cancer, reorganization of genome fragments results in new aberrant chimeric genes as a product of the fusion of the fragments, found less frequently in solid tumors (257). However, in primary gastric tumors and cell lines of gastric cancer and in primary colorectal cancer, the fusion of coding region of SLC1A2 (EAAT2) with a region of a probable promoter of CD44 results in a truncated but overexpressed and functional protein EAAT2 (189, 258). This truncated EAAT2 contributes to augmented glutamate concentrations that regulate the growth and development of gastric cancer (189). Furthermore, CD44-SLC1A2 gene silencing sensitizes gastric cancer cells to chemotherapy with cisplatin (189) which provides an additional target for therapy intervention.

Although more evidence is necessary for establishing the role of EAATs in the development and progression of cancer, here is another potential target for cancer therapy that includes selective inhibition of EAATs.

The use of drugs targeting glutamate receptors and/or transporters in combination with approved therapies could offer a more specific scheme of treatment with fewer adverse effects.

## 5 Concluding remarks

**Figure 4:**
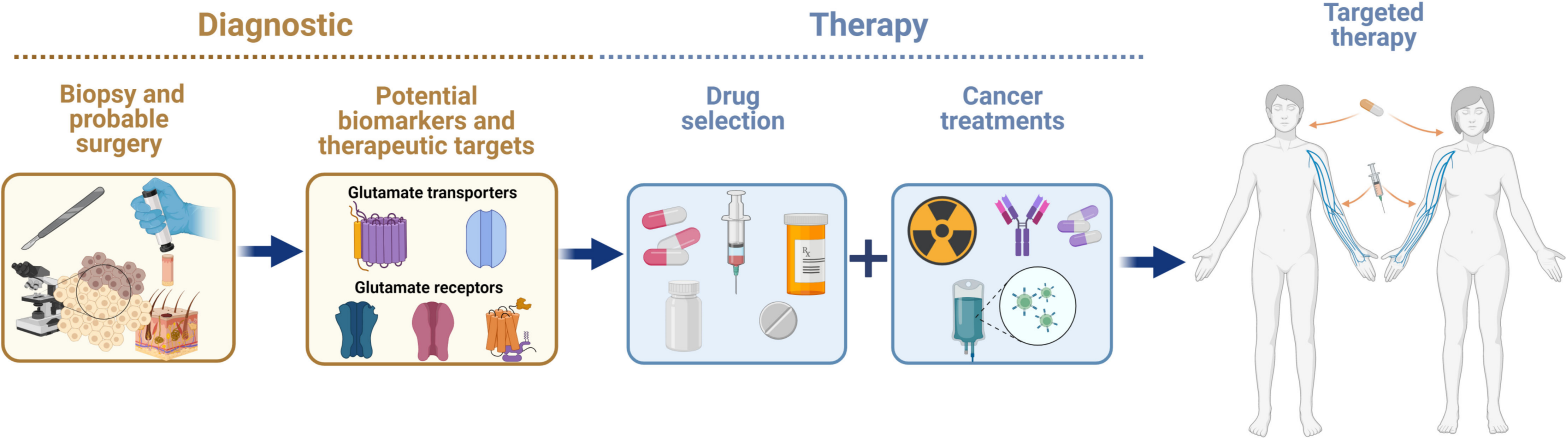
Advances in knowledge on the expression and actions of glutamatergic system components in cancer in non-neural organs have allowed researchers to consider them as potential biomarkers and therapeutic targets for alleviating the effects of cancer therapy.

The identification and validation of biomarkers in early stages of cancer, as well as more specific therapeutic targets, is still a challenge for cancer research. The heterogenous nature of cancer makes it difficult to provide a prompt diagnosis and find a specific therapy to improve the prognosis of patients with cancer. Pharmacological approaches using receptor agonists or antagonists and transporter inhibitors, or molecular biology approaches, such as knock out, knock in, or silencing of gene expression, have been used to demonstrate that the glutamatergic system has an important role in oncogenesis. Glutamatergic system components are differentially expressed in non-neural organs in cancer and healthy organs, suggesting that this system supports cancer progression. In this regard, glutamate agonists or antagonists, glutamate transporter inhibitors, and molecular strategies could be used as powerful tools to interfere the mechanism of action of glutamatergic system components in order to treat malignant tumors. A variety of cancers depend on xCT overexpression for survival. Blocking of xCT could avoid the progression of a variety of cancer hallmarks by inhibiting protection against ROS overproduction and/or impeding its glutamate exporting activity and the subsequent binding and activation of glutamate receptors (**Figure 3**). For example, breast cancer has been well studied in which glutamate overproduction by enhanced glutaminase is released *via* the system xCT to activate mGluR3 signaling to increase invasiveness, and xCT inhibition with sulfasalazine decreases breast tumor growth. Besides, concomitant use of these drugs with other approved treatments may be helpful to eliminate tumors on multiple fronts. Several studies have highlighted the potential of combined treatment approaches as more effective therapeutic strategies than a single treatment for cancer. Hence, designing novel combinations of drugs that trigger glutamatergic system components, taking advantage of those with fewer side effects that do not penetrate the blood-brain barrier seems to be feasible. The efficacy and safety of combining drugs that include glutamatergic system components for clinical use requires further research that considers the expression pattern of the elements of the glutamatergic system (**Figure 4**).

## Author contributions

IM provided critical advice during the writing. MD-M. critically revised the content. AH-A performed the creative design of the figures. All authors contributed to the writing of the article and approved the submitted version.

## Funding

This work was supported by CONACyT, México (239250 for IM), PAPIIT-DGAPA, UNAM México (IN206418 and IN222821 for IM) (IN202121 for MD-M).

## Acknowledgments

We thank Jessica Norris for critically editing the manuscript. AG-G is a Doctoral student from the Programa de Posgrado en Ciencias Biomédicas, Universidad Nacional Autónoma de México (UNAM). AH-A is a Master student from Programa de Posgrado en Ciencias Bioquímicas, Universidad Nacional Autónoma de México (UNAM). Both students received fellowships from Mexico’s National Council of Science and Technology (CONACyT) (AH-A, scholarship 1084428).

## Conflict of interest

The authors declare that the research was conducted in the absence of any commercial or financial relationships that could be construed as a potential conflict of interest.

## Publisher’s note

All claims expressed in this article are solely those of the authors and do not necessarily represent those of their affiliated organizations, or those of the publisher, the editors and the reviewers. Any product that may be evaluated in this article, or claim that may be made by its manufacturer, is not guaranteed or endorsed by the publisher.
